# Ultrasound-Assisted Dispersive Liquid-Liquid Microextraction Using Deep Eutectic Solvents (DESs) for Neutral Red Dye Spectrophotometric Determination

**DOI:** 10.3390/molecules27186112

**Published:** 2022-09-19

**Authors:** Sana Ullah, Hameed Ul Haq, Muhammad Salman, Faheem Jan, Faisal Safi, Muhammad Balal Arain, Muhammad Shahzeb Khan, Roberto Castro-Muñoz, Grzegorz Boczkaj

**Affiliations:** 1 Department of Chemistry, University of Malakand, Chakdara 18800, Pakistan; 2 Department of Sanitary Engineering, Faculty of Civil and Environmental Engineering, Gdansk University of Technology, G. Narutowicza St. 11/12, 80-233 Gdansk, Poland; 3 School of Materials Science and Engineering, University of Science and Technology of China, Shenyang 110016, China; 4 Department of Advanced Materials Center, Faculty of Electronics, Telecommunications and Informatics, Gdansk University of Technology, G. Narutowicza St. 11/12, 80-233 Gdansk, Poland; 5 Department of Chemistry, University of Karachi, Karachi 75270, Pakistan; 6 Department of Chemistry and Technology of Functional Materials, Faculty of Chemistry, Gdansk University of Technology, G. Narutowicza St. 11/12, 80-233 Gdansk, Poland; 7 Tecnologico de Monterrey Campus Toluca, Av. Eduardo Monroy Cárdenas 2000 San Antonio Buenavista, Toluca de Lerdo 50110, Mexico; 8 EkoTech Center, Gdansk University of Technology, G. Narutowicza St. 11/12, 80-233 Gdansk, Poland

**Keywords:** neutral red dye, deep eutectic solvents (DES), sample preparation, extraction, spectrophotometric analysis

## Abstract

Deep eutectic solvents (DES), which have low toxicity and are low cost, biodegradable, and easily synthesized, were used for the extraction of neutral red (NR) dye before its spectrophotometric analysis. DES, containing choline chloride as a hydrogen bond acceptor and phenol as a hydrogen bond donor with a molar ratio of 1:2, was used for the extraction of NR dye from aqueous media. The possible interaction of different DESs with NR was studied using density functional theory (DFT) calculations. Experimentally, a UV-visible spectrophotometer was used for the quantitative analysis. The most important parameters affecting method performance, such as pH, extraction temperature, DES type, its volume, THF volume, sonication time, and centrifugation time, were optimized. The developed method provides exceptional sensitivity in terms of LOD and LOQ, which were 2.2 and 7.3 µg/L respectively. The relative standard deviation was 1.35–1.5% (*n* = 10), and the pre-concentration factor was 40. The method was found to be linear in the range of 2–300 µg/L (R2 = 0.9967). The method was successfully used for the determination of NR in wastewater samples. Finally, the DES-based method presents operational simplicity, high sensitivity, and rapid determination (<5 min) compared with other analytical procedures.

## 1. Introduction

According to the literature, approximately 0.7 million tons of 10,000 various dyes and pigments are annually synthesized. It is estimated that 2–5% of them have been found as pollutants in the wastewater discharged from various industries, such as leather, textile, cosmetics, glass, food, etc.; hence, this is alarming to the low quality of the environment. Furthermore, less than 1 ppm of them is observable and objectionable in water [1,2,3,4]. Dyes are one of the most poisonous and hazardous substances which affect photosynthesis in aquatic plants and also enhance the biological oxygen demand. Furthermore, some health issues in humans, such as skin irritation, abnormal liver function, and kidney failure, have been attributed to such substances [5,6].

For instance, neutral red (NR) (according to IUPAC-*hydrochloride 3-amino-6-dimethylamino-2 methyl phenazine*) is a cationic dye in the form of an azine derivative [7]. NR dye also has many other applications, such as colorants for cotton, wool, silk, etc. [8]. In biological research, NR is used as a coloring reagent for embryonic tissues and other living materials. Besides the above-mentioned toxic effects, NR has a unique toxic effect, as it can enter the 56 body cells and become accumulated in the cytoplasm [9,10].

The industrial wastes containing these dyes, which are discharged into the water bodies without any pretreatment, can adversely affect the aquatic ecosystem and human health [11,12]. Due to their above-mentioned harmful effects on the environment and humans, it is necessary to remove them from water; however, due to their high resistance to oxidation and light, it is difficult to remove them from aqueous streams [13]. Various analytical techniques used for the determination of dyes in water include liquid chromatography-mass spectrometry (LC-MS/MS) [14], gas chromatography-mass spectrometry (GC-MS) [15], high-performance liquid chromatography [16], and thin-layer chromatography [17]. As for dye analysis, UV-Vis spectrophotometry is among the most commonly used analytical techniques, as it is relatively robust, rapid, cheap, accurate, and precise compared to the other mentioned techniques [18,19]. The main issue associated with UV-Vis spectrophotometry is its relatively low selectivity for direct determination when the sample contains traces of the analyte. It is known that the interfering species in the matrix interact with the analyte and thus affect its signal [20].

Liquid-liquid extraction is considered one of the effective techniques for the preparation of aqueous dye samples for analysis [21]. Unfortunately, its use has been dispirited due to its longer extraction time, toxic nature, and the large volumes of organic solvent which are required [22,23]. In this method, deep eutectic solvents (DESs) have been used for NR pre-concentration. DESs are solutions of Lewis or Brønsted acids and bases which form eutectic mixtures. DESs are considered tunable solvents through varying the structure or molar ratio of components and thus have a wide variety of potential applications [24]. The components of DES can interact with each other in a complex hydrogen-bonding network, resulting in a significant decrease in the melting point [25]. Generally, DESs are characterized by a significantly large reduction in their melting point compared to their pure components [26,27]. DESs are also recognized for their green properties (non-toxicity, complete biodegradability, and biocompatibility), extraction properties, and low cost [28,29,30,31,32]. The latter characteristics have attracted the attention of the research community.

Due to the disadvantages of the above-mentioned methods, we developed a new assay for the simultaneous extraction of NR from water by using a DES system. This method aims to be highly effective, requiring less time as well as lowering the consumption and costs of chemicals.

## 2. Results and Discussions

### 2.1. Optimization of pH during Extraction

**Figure 1:**
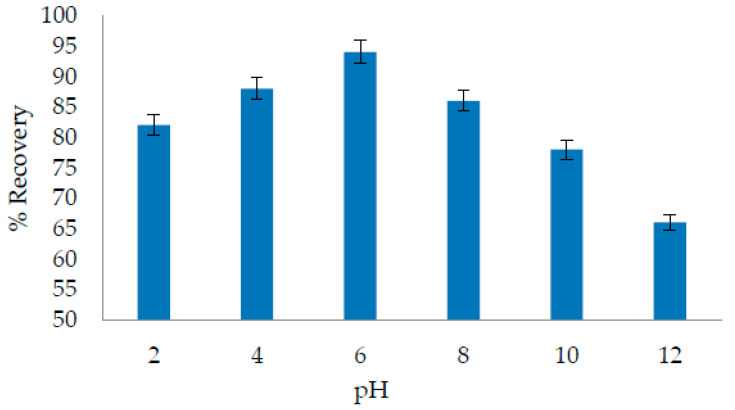
Optimization of pH. Buffer volume-2 mL; sample volume-20 mL; analyte concentration-20 µg/L; DES volume-500 µL; THF volume-300 µL; temperature-25 °C; sonication time-2 min; centrifugation time-1 min.

NR is used as a pH indicator. In acidic media, it has a red color, while in basic media above pH 7, it has a yellow color. The NR dye extraction is sensitive to pH conditions. In the DES-based extraction method, the pH of the solution can affect the interaction of the analyte with the DES [33]. In addition, pH affects the extraction efficiency of DESs [29,32]. To study the effect of pH, six different buffer solutions were prepared with a pH range of 2–12. Figure 1 shows the results for pH optimization. The maximum extraction efficiency was observed at pH 6. A moderate optimum pH makes this method more favorable because it does not require large amounts of strong acid or a base to adjust the pH. A turbid cloudy solution was observed at pH 12 with no clear separation of the DES layer. All the experiments were performed at room temperature (25 °C).

### 2.2. Solvent Selection and Optimization of Extraction Conditions

**Figure 2:**
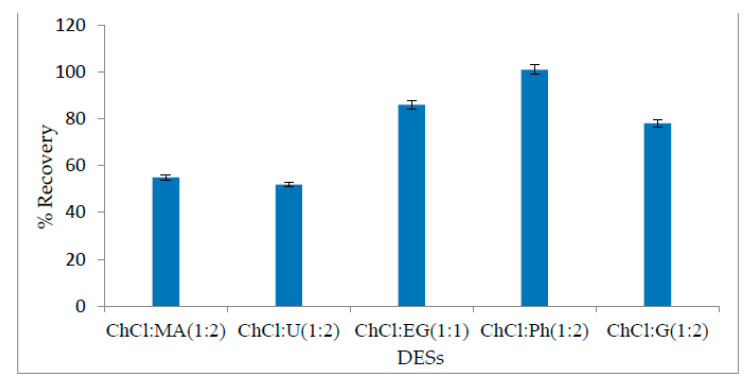
Selection of the DES for maximum % recovery. Analyte concentration-20 µg/L; buffer volume-2 mL (pH = 6); sample volume-20 mL; DES volume-500 µL; THF volume-300 µL; temperature-25 °C; sonication time-2 min; centrifugation time-1 min.

The selection of a suitable solvent is of prime importance in an analytical method. The selection criteria for a suitable solvent are based on its cost-effectiveness, availability, high selectivity, and percentage of recovery during analyte extraction. In this method, a new class of organic solvents, “deep eutectic solvents”, was selected for this method. Six different types of DESs were tested for NR extraction with their optimum molar ratio at different pHs. The percentages of recovery for different DESs at their optimum pH are shown in Figure 2. The DES based on choline chloride and ethylene glycol (1:1) gave 86% recovery, while maximum recovery (101%) was obtained from a DES based on choline chloride (ChCl) and phenol (Ph) (1:2). Even if both DESs can be used for NR extraction, the ChCl + ethylene, glycol-based DES is more eco-friendly; however, its % recovery is comparatively lower. On the other hand, DESs based on choline chloride + phenol offered maximum % recovery. Some researchers have raised objections over the use of ChCl-Ph-based DESs for extraction. Shishov et al. reported changes in the composition of ChCl-Ph in the aqueous medium in the presence of THF [34]. This is an important aspect for further studies of DES fundamentals. In the current paper, we confirmed that it is possible to obtain reproducible results and linearity from the calibration curve using ChCl-Ph. Even if the composition of the DES phase was changed during the extraction, it was still proved that the developed method fully fitted the purpose.

The volume of DES has been optimized in the range of 0.1 mL to 0.6 mL. By increasing the volume of DES from 0.1 mL to 0.5 mL, a gradual increase was observed in the % recovery of NR. Further increases in the DES volume resulted in the dilution of the analyte, and a slight decrease in % recovery was observed. Therefore, 0.5 mL was selected as the optimum volume for 25 µg/L NR in a 20 mL sample. Figure S1 shows the results of the DES volume optimization. The optimum volume of THF was determined for a 20 mL sample with an NR concentration of 400 µg/L. The results for THF optimization are shown in Figure S2 (See Supplementary Materials).

The time for sonication and centrifugation was also optimized. As for the time of sonication, it varied from 0.25 to 4 min, and it was found that 2 min is sufficient for sonication. The optimum time for centrifugation was determined by changing the centrifugation time from 0.25 to 4 min, and a maximum recovery was obtained for 1 min; therefore, this time was selected as the optimum time for centrifugation. The results for sonication time and centrifugation time are described in Figure S3 (See Supplementary Materials).

### 2.3. Analytical Performance of the Developed Method

**Figure 3:**
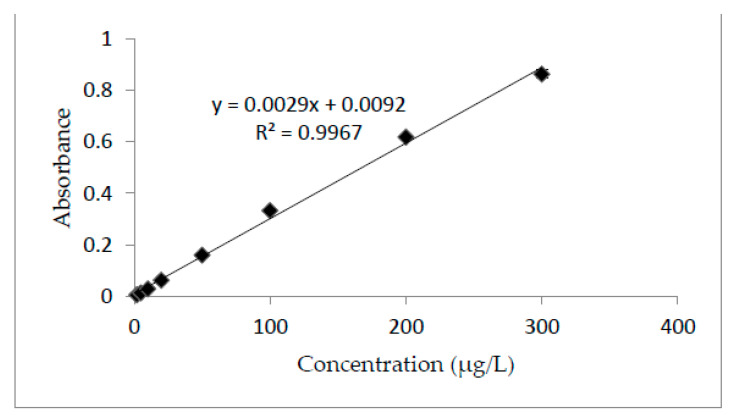
Calibration curve. Analyte concentration-2–300 µg/L; buffer volume-2 mL (pH = 6); sample volume-20 mL; DES volume-500 µL; THF volume-300 µL; temperature-25 °C; sonication time-2 min; centrifugation time-1 min.

All analytical parameters were determined according to the standard procedures reported in the literature [35,36,37]. Under optimal conditions, the % recovery was found to be as high as 101.95–102.9%. The pre-concentration factor was found at 40, while the limit of detection and limit of quantification were about 2.20 µg/L and 7.34 µg/L, respectively, confirming that the developed assay fits the purpose, as well as providing satisfactory sensitivity for the analysis of environmental samples. This method is robust and allows precise results to be obtained. The linearity of the method was confirmed for a range of concentrations of 2–300 µg/L. The method was found to be linear from a low to high concentration following Beer-Lambert law. The linear curve equation for the calibration curve was y = 0.0029x + 0.0092, with a coefficient determination (R2) of 0.9967. For absorbance (y), concentration (x) was calculated in the real sample. Figure 3 shows the results for the standard curve.

### 2.4. Method Application

Since typical sources of NR are the wastewaters from various industries, such as leather, textile, cosmetics, glass, and food, this method was used for the analysis of NR in wastewater samples. The standard addition method was used to decrease the effect of interfering species. Triplicate composite samples were analyzed using a UV-visible spectrophotometer. The samples were spiked with standard analyte concentrations (5 µg/L, 10 µg/L and 20 µg/L). NR concentration in the real sample was less than LOD (2.2 µg/L). The % recovery of analyte in the real samples was 101.95–102.9%, with RSD at 1.0–1.8%. The results of this study are presented in Table 1.

### 2.5. Comparative Study

The newly developed method was compared with the reported method in the literature.

To the best of our knowledge, few analytical methods are available for the analysis of NR. A comparative study of the developed method with already reported methods in the literature is presented in Table 2.

Wang Shi-Ru et al. reported a method for the extraction and analysis of NR based on ionic liquids [41]. However, the use of ionic liquids could have an extremely negative impact on the environment due to their relatively high toxicity and poor biodegradability [42]. Furthermore, the extraction time for this method is more than 28 min, while the extraction time estimated for the developed method is 5.5 min. Another HPLC-based method was developed for NR analysis using ionic liquids as eluent additives; however, this method is less sensitive than our reported method, although it requires advanced instrumentation. Furthermore, the use of ionic liquids makes this method non-eco-friendly.

Another approach is based on the pre-concentration method for NR dye, in which functionalized–multiwalled carbon nanotubes using atmospheric-pressure matrix-assisted laser desorption/ionization mass spectrometry (AP-MALDI/MS) has been reported [40]. This latter method involves the use of expensive chemicals, complicated preparation, and complicated instrumentation. Additionally, this method requires a longer extraction time (≥50 min) and lower % recovery.

Moawed et al. reported a spectrophotometric method for NR analysis [39], which was only applicable at the mg/L level and required more toxic chemicals. So far, it is clear that only a few methods are available for the pre-concentration and extraction of NR dye (see Table 2). There is no single solvent available to directly extract NR dye. In general, these methods involve the application of sophisticated items for sample preparation, such as hollow fibers and functionalized–multiwalled carbon nanotubes, as shown in the comparative table. Interestingly, our developed method is much faster compared to other methods reported in the literature. For instance, the time required for sample preparation in this method is less than 5.5 min, while for other methods; the estimated time was at least 28 min. Furthermore, our method has the highest value for the enrichment factor (40), which makes this method applicable for NR determination at very low concentrations. It is worth mentioning that the developed method is also much more robust compared to other methods based on hollow fibers or carbon nanotubes, as it is based on a simple extraction system, opening up the possibility to be implemented in any laboratory for water and wastewater analysis.

In contrast to the above-mentioned methods, this new method is highly sensitive, faster, easier, uses greener solvents, and is simple because it does not require any complicated instrumentation. This new method does not require any heat and gives 100% efficiency at room temperature. The only disadvantage of this method is the use of phenol in DES, which causes the method to not fulfill all the principles of green chemistry.

### 2.6. Computational Study of DES and NR Interaction

The density-functional theory (DFT) calculations were carried out with the Gaussian g09 package. All the structures were fully optimized with hybrid functional B3LYP [43] with a 6-311++G (d,p) basis set [44]. The vibrational analysis confirms the true minima with no imaginary frequency at the same level as the density functional theory method. In the case of intermolecular forces, Grimme’s D3 method was included in the B3LYP/6-311++G (d,p) calculation [45].

**Figure 4:**
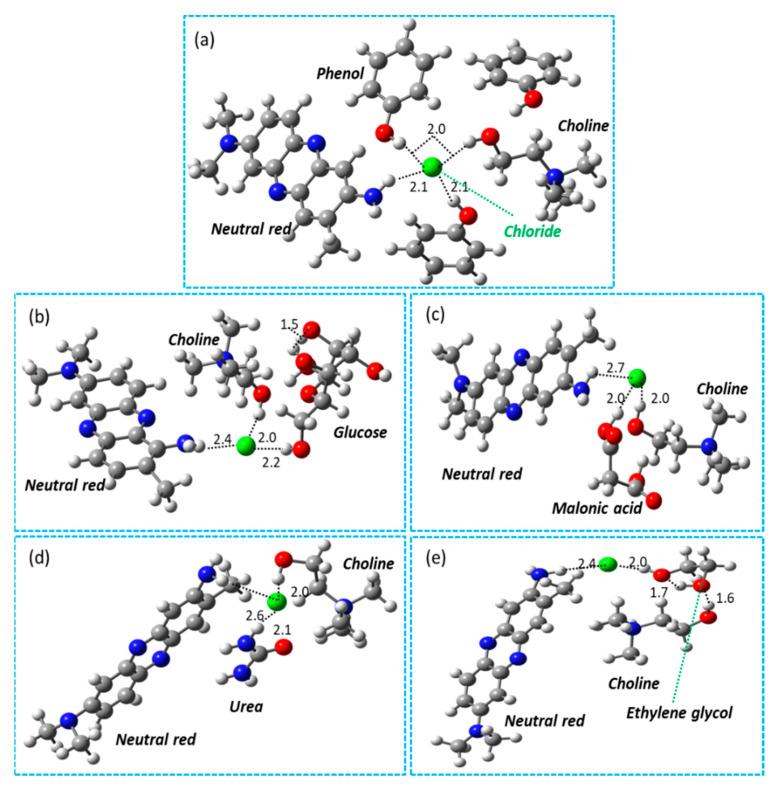
Representation of interaction between different DESs and NR molecules using density-functional theory (DFT) calculations. (**a**) DES (choline chloride + phenol) interaction with NR. (**b**) DES (choline chloride + glucose) interaction with NR. (**c**) DES (choline chloride + malonic acid) interaction with NR. (**d**) DES (choline chloride + urea) interaction with NR. (**e**) DES (choline chloride + ethylene glycol) interaction with NR. The gray, red, blue, green, and white colors represent the carbon, oxygen, nitrogen, chloride and hydrogen species, respectively.

The possible hydrogen bonding between DESs and NR is calculated, as shown in Figure 4a–e. Computational studies show that the Cl- of DES interacts with NR molecules. The results in Figure 4a–e clearly show a stronger interaction of DES4 (Choline chloride + Phenol (1:2)) with NR molecules with a shorter bond length of 2.1 Å. Cl- has a longer bond length in glucose (2.4 Å), malonic acid (2.7 Å), urea (2.6 Å), and ethylene glycol (2.4 Å) than in other DESs. Furthermore, the results show that the interaction between OH groups is stronger and is estimated as 1.6 Å. By contrast, the interaction between NH-Cl- and OH-Cl- is found in a range of 2.0 Å, as shown in Figure 4. In the case of NR, choline chloride, and phenol species, a network of hydrogen bonding is found between -OH-OH-, OH-Cl-, and NH-Cl-, as shown in Figure 4a. The Cl- anions are surrounded by the NH group of NR, the OH group of choline, and phenol molecules. On this basis, it can be concluded that more hydrogen bonding is advantageous for DES-assisted extraction.

## 3. Materials and Methods

### 3.1. Chemicals and Reagents

The used chemicals were: ethanol, NR dye, choline chloride, phenol, tetrahydrofuran, acetic acid, ammonium chloride, ammonium hydroxide, ammonium acetate, ammonia, phosphoric acid, hydrated sodium dihydrogen phosphate, and deionized water. All the chemicals were purchased from Sigma Aldrich (Hamburg, Germany) and were used without further purification. The stock solution of 500 ppm of NR standard was prepared in water. A 25 ppm NR standard solution was prepared from the stock solution by dilution using deionized water. The buffer solutions, which maintained the pH as 2 and 4, were prepared using phosphoric acid and hydrated sodium dihydrogen phosphate, respectively. A pH 6 buffer was prepared from acetic acid and ammonium acetate. A pH of 8–12 was obtained using ammonium chloride and ammonium hydroxide. Finally, the pH of the buffer solutions was adjusted using a pH meter. For experimental analysis, the wastewater sample was taken from Mardan Sugar Mill (Mardan, Pakistan).

### 3.2. Instrumentation

The UV-visible spectrophotometer model Shimadzu UV-120-02 (Burladingen, Germany) was used to measure the absorbance of the samples. For mixing and centrifugation processes, the HAPA ultrasonic bath (Shenzhen, China) and Hettich EBA 20 (Darmstadt, Germany) centrifuge was used. Furthermore, the pH was measured by using the HI 2211 pH/ORP meter (Darmstadt, Germany). For water deionization, a deionizer model LP-15/30/45 (FINETECH water treatment technologies, Karachi, Pakistan) was used.

### 3.3. DES Preparation

Five different types of choline-chloride-based DESs were selected for NR extraction. Herein, five different HBDs were selected based on their functional group. Choline chloride is hygroscopic; thus, it was heated at 80 °C for 120 min to remove the possible water contents. DES1 was prepared by mixing choline chloride and malonic acid with a molar ratio of 1:2, while DES2 was prepared by mixing choline chloride and urea with a molar ratio of 1:2. DES3 was prepared by mixing choline chloride and ethylene glycol with a molar ratio of 1:1. DES4 was prepared by mixing choline chloride and phenol with a molar ratio of 1:2. DES5 was prepared by mixing choline chloride and glucose with a molar ratio of 1:2. All DESs were treated under similar conditions and mixed under magnetic stirring at 60 °C for 10 min. Finally, homogenous mixtures were obtained in the form of DESs, which were further used for optimization studies.

### 3.4. Sample Preparation and Extraction Experiments

The wastewater samples were collected in black plastic bottles from the stream of a small industrial state in Mardan. The samples were collected from 5 different locations, each with an intermediate distance of approximately 200 m. Triplicate samples were collected for each sampling point. All the samples were filtered with a GV syringe filter (0.22 μm Sterile Hydrophilic PVDF, 13 mm). The typical sample used in the experiments had a volume of 20 mL. A 2 mL buffer of pH 6 was added to each sample. Furthermore, 0.5 mL of selected DES was added to the mixture and sonicated for 2 min. An amount of 0.4 mL of demulsifying agent was added to enhance the analyte recovery. The sample was centrifuged for 1 min at a speed of 4000 rpm for layer separation. The upper DES layer was easily separable. The DES layer containing NR was extracted through a syringe filter and added to a clean Falcon tube. After this, it was diluted up to 3 mL with ethanol, and then the absorbance was determined through a UV-visible spectrophotometer. Ethanol was used as a blank solution in a UV-visible spectrophotometer.

### 3.5. Determination of Absorption Peak (λ_max_)

NR dye has a maximal absorption of light (λ_max_) at 530 nm already reported in the literature. However, this value could be influenced by several operating conditions, such as the pH of the solution [46] and the type and composition of DES [47]. To determine the λ_max_ for the extracted layer, the absorbance was scanned from 370 nm to 800 nm. A spectral shift was observed in the presence of the buffer solution as well as DES. Maximal absorbance at pH 6 with a DES made of choline chloride and phenol (1:2) was observed at 460 nm; therefore, this wavelength was selected as λ_max_. The results for determining the λ_max_ are shown in Figure S4 (See Supplementary Materials).

### 3.6. Calculation of Percentage of Recovery and Validation Assays

Percentage of recovery (%R) was evaluated as a reference to determine the appropriate values of extraction parameters in the optimization studies. %R was calculated according to the following equation:

%R = Cd/Ce × 100(1)

where Cd is the concentration determined in the spiked real sample and Ce is the expected concentration in the spiked real sample. Spiked samples were prepared by adding the different volumes of standard NR solution to the wastewater samples with final concentrations of 5 µg/L, 10 µg/L and 20 µg/L.

The residual standard deviation was calculated for the regression line using excel. According to the AOAC-Association of Analytical Communities-LOD can be defined as the “Lowest content that can be measured with reasonable statistical certainty”, while LOQ is the lowest amount of analyte in a sample, which can be quantitatively determined with suitable precision and accuracy [48]. Based on the standard deviation of the slope, LOD and LOQ can be estimated using the following equations:

LOD = (3 × SD)/m(2)

LOQ = (10 × SD)/m(3)

where LOD is the limit of detection; SD is the residual standard deviation of regression lines; m is the slope of the calibration curve; and LOQ is the limit of quantification [49]. The relative standard deviation was calculated using the following equation:

RSD (%) = SD/Cm × 100(4)

In the above equation, SD represents the residual standard deviation, while Cm is the mean concentration of the analyte.

The pre-concentration factor (PF) can be calculated as “The concentration ratio of the analyte in the final extract (DES phase) ready for its determination and in the initial solution” [50]. The pre-concentration factor was evaluated by using the following equation [51]:

PF = Cf/Ci(5)

where Cf and Ci are the final concentrations and initial concentrations of analytes in the DES phase (receiving phase) and donor phase, respectively.

## 4. Conclusions

In this work, a new analytical method has been developed for the analysis of NR dyes in wastewater. Deep eutectic solvents based on ChCl and phenol with a 1:2 molar ratio demonstrated a maximum recovery for NR extraction. In conclusion, NR dyes can be successfully extracted from aqueous mediums by using deep eutectic solvents, which are considered a new class of green organic solvents due to their low cost, biodegradability, easy preparation, limited toxicity, and the wide availability of needed chemicals. The percentage of recovery (101–102%) for the spiked samples demonstrated the suitability of the optimized method. The LOD and LOQ were 2.2 µg/L and 7.3 µg/L, respectively, with an RSD value of 1.35–1.5% and an enrichment factor of 40. The comparison of the developed method with already-existing analytical procedures confirmed its advantages, such as shortening the time of analysis, robustness, sensitivity, simple instrumentation, and eco-friendly character. To the best of our knowledge, this is a pioneering DES-based method for the analysis of NR. Finally, it can be concluded that this method can be used for NR analysis in wastewater samples at µg/L.

## Figures and Tables

**Table 1:** Application of the method for NR in wastewater (industrial effluents).

Sample	Analyte Added (µg/L)	Analyte Found (µg/L)	% Recovery	% RSD (*n* = 3)
Wastewater (Industrial effluents)	0.00	<LOD		
	5	5.11	102.2	±1.0
	10	10.29	102.9	±1.5
	20	20.39	101.95	±1.8

**Table 2:** Comparison of analytical methods and parameters for NR.

Analytical Method	LOD (µg/L)	RSD (%)	Linearity Range (µg/L)	% Recovery	EF	Estimated Time (min)	Samples Application	Reference
^a^ HFLPME/^b^ HPLC	0.3	4.3	1.0–10.0	95.0–112.0	00	≥28	Standard only	[38]
^c^ FMWCNT/^d^ UV-SP	1.0	12.3	12–40	88.6–98.4	---	≥40	Wastewater	[39]
^e^ FMCN/^f^ AP-MALDI/MS	4.47	0.14			20–32	≥50	River water	[40]
^g^ IL-HFLPME/^d^ UV-SP	100	1.5		96.4	15	≥33	Soft drinks	[41]
^h^ DES-LPME/^d^ UV-SP	2.2	1.5	2–400	102	40	≤5.5	Wastewater	This work

^a^ HFLPME-hollow fiber liquid phase microextraction; ^b^ HPLC-high performance liquid chromtography; ^c^ FMWCNT-functionalized–multiwalled carbon nanotubes; ^d^ UV-SP-ultraviolet visible spectrophotometric; ^e^ FMCN-functionalized–multiwalled carbon nanotubes; ^f^ AP-MALDI/MS-atmospheric pressure matrix assisted laser desorption/ionization mass spectroetry; ^g^ IL-HFLPME-ionic liquids hollow-fiber liquid phase microextraction; ^h^ DES-LPME-deep eutectic solvent based liquid phase microextraction.

## Data Availability

Not applicable.

## Supplementary Materials

The following supporting information can be downloaded at: https://www.mdpi.com/article/10.3390/molecules27186112/s1, Figure S1: Optimization of DES volume, THF volume: 300µL, Sample volume: 20 mL, NR concentration: 400 µg/L; Figure S2: Optimization of THF volume, DES volume 500 µL, Sample volume: 20 mL, NR concentration: 400 µg/L; Figure S3: Time optimization for sonication and centrifugation, DES volume 500 µL, THF volume: 300µL, Sample volume: 20 mL, NR concentration: 400 µg/L; Figure S4: Selection of optimum wavelength (Lambda max) for NR quantification.

## Sample Availability

Samples of the compounds and DESs are available from the authors (H.U.H. and G.B.).

## References

[1] Gupta V.K., Jain R., Nayak A., Agarwal S., Shrivastava M. (2011). Removal of the hazardous dye—Tartrazine by photodegradation on titanium dioxide surface. Mater. Sci. Eng. C.

[2] Kobya M., Gengec E., Demirbas E. (2016). Operating parameters and costs assessments of a real dyehouse wastewater effluent treated by a continuous electrocoagulation process. Chem. Eng. Process. Process Intensif..

[3] Zhang W., Liu W., Zhang J., Zhao H., Zhang Y., Quan X., Jin Y. (2012). Characterisation of acute toxicity, genotoxicity and oxidative stress posed by textile effluent on zebrafish. J. Environ. Sci..

[4] Adegoke K.A., Bello O.S. (2015). Dye sequestration using agricultural wastes as adsorbents. Water Resour. Ind..

[5] Zhu T., Chen J.S., Lou X.W. (2012). Highly efficient removal of organic dyes from waste water using hierarchical NiO spheres with high surface area. J. Phys. Chem. C.

[6] Cako E., Gunasekaran K.D., Soltani R.D.C., Boczkaj G. (2020). Ultrafast degradation of brilliant cresyl blue under hydrodynamic cavitation based advanced oxidation processes (AOPs). Water Resour. Ind..

[7] Stec A.A., Hull T.R. (2010). Fire Toxicity.

[8] Deng H., Lu J., Li G., Zhang G., Wang X. (2011). Adsorption of methylene blue on adsorbent materials produced from cotton stalk. Chem. Eng. J..

[9] Oseroff A., Ohuoha D., Ara G., McAuliffe D., Foley J., Cincotta L. (1986). Intramitochondrial dyes allow selective in vitro photolysis of carcinoma cells. Proc. Natl. Acad. Sci. USA.

[10] Bayramoglu G., Altintas B., Arica M.Y. (2009). Adsorption kinetics and thermodynamic parameters of cationic dyes from aqueous solutions by using a new strong cation-exchange resin. Chem. Eng. J..

[11] Comm C., Energy J.M.P.E., Dev O.P.R. (2008). Publication list. Org. Process. Res. Dev.

[12] Bulgarelli D.L., Ting A.Y., Gordon B.J., de Sá Rosa A.C.J., Zelinski M.B. (2018). Development of macaque secondary follicles exposed to neutral red prior to 3-dimensional culture. J. Assist. Reprod. Genet..

[13] Baig U., Uddin M.K., Gondal M. (2020). Removal of hazardous azo dye from water using synthetic nano adsorbent: Facile synthesis, characterization, adsorption, regeneration and design of experiments. Colloids Surf. A: Physicochem. Eng. Asp..

[14] Guerra E., Celeiro M., Lamas J.P., Llompart M., Garcia-Jares C. (2015). Determination of dyes in cosmetic products by micro-matrix solid phase dispersion and liquid chromatography coupled to tandem mass spectrometry. J. Chromatogr. A.

[15] Balçık U., Chormey D.S., Ayyıldız M.F., Bakırdere S. (2020). Liquid phase microextraction based sensitive analytical strategy for the determination of 22 hazardous aromatic amine products of azo dyes in wastewater and tap water samples by GC-MS system. Microchem. J..

[16] Lian Z., Wang J. (2012). Molecularly imprinted polymer for selective extraction of malachite green from seawater and seafood coupled with high-performance liquid chromatographic determination. Mar. Pollut. Bull..

[17] Ai M. (1978). Catalytic activity for the oxidation of methanol and the acid-base properties of metal oxides. J. Catal..

[18] Guo Y., liu C., Ye R., Duan Q. (2020). Advances on Water Quality Detection by UV-Vis Spectroscopy. Appl. Sci..

[19] Mabood F., Hussain Z., Haq H., Arian M., Boqué R., Khan K., Hussain K., Jabeen F., Hussain J., Ahmed M. (2016). Development of new UV–vis spectroscopic microwave-assisted method for determination of glucose in pharmaceutical samples. Spectrochim. Acta Part A: Mol. Biomol. Spectrosc..

[20] Hashemi S.H., Kaykhaii M., Keikha A.J., Sajjadi Z. (2018). Application of Box-Behnken design in response surface methodology for the molecularly imprinted polymer pipette-tip solid phase extraction of methyl red from seawater samples and its determination by spectrophotometery. Mar. Pollut. Bull..

[21] Schweitzer P.A. (1979). Handbook of Separation Techniques for Chemical Engineers.

[22] Ferreira A.M., Coutinho J.A., Fernandes A.M., Freire M.G. (2014). Complete removal of textile dyes from aqueous media using ionic-liquid-based aqueous two-phase systems. Sep. Purif. Technol..

[23] Rydberg J., Musikas C., Choppin G.R. (1992). Principles and Practices of Solvent Extraction.

[24] Smith L.E., Abbott A.P., Ryder K.S. (2014). Deep eutectic solvents (DESs) and their applications. Chem. Rev..

[25] Abbott A. (2010). Deep Eutectic Solvents.

[26] Tang B., Zhang H., Row K.H. (2015). Application of deep eutectic solvents in the extraction and separation of target compounds from various samples. J. Sep. Sci..

[27] Florindo C., Oliveira F.S., Rebelo L.P.N., Fernandes A.M., Marrucho I.M. (2014). Insights into the synthesis and properties of deep eutectic solvents based on cholinium chloride and carboxylic acids. ACS Sustain. Chem. Eng..

[28] Abbott A.P., Capper G., Davies D.L., Rasheed R.K., Tambyrajah V. (2003). Novel solvent properties of choline chloride/urea mixtures. Chem. Commun..

[29] Haq H.U., Balal M., Castro-Muñoz R., Hussain Z., Safi F., Ullah S., Boczkaj G. (2021). Deep eutectic solvents based assay for extraction and determination of zinc in fish and eel samples using FAAS. J. Mol. Liq..

[30] Marchel M., Cieśliński H., Boczkaj G. (2022). Deep eutectic solvents microbial toxicity: Current state of art and critical evaluation of testing methods. J. Hazard. Mater..

[31] Haq H.U., Bibi R., Arain M.B., Safi F., Ullah S., Castro-Muñoz R., Boczkaj G. (2022). Deep eutectic solvent (DES) with silver nanoparticles (Ag-NPs) based assay for analysis of lead (II) in edible oils. Food Chem..

[32] Faraz N., Haq H.U., Arain M.B., Castro-Muñoz R., Boczkaj G., Khan A. (2021). Deep eutectic solvent based method for analysis of Niclosamide in pharmaceutical and wastewater samples–A green analytical chemistry approach. J. Mol. Liq..

[33] Faraji M., Mahmoodi-Maymand M., Dastmalchi F. (2020). Green, fast and simple dispersive liquid-liquid microextraction method by using hydrophobic deep eutectic solvent for analysis of folic acid in fortified flour samples before liquid chromatography determination. Food Chem..

[34] Shishov A., Gorbunov A., Moskvin L., Bulatov A. (2020). Decomposition of deep eutectic solvents based on choline chloride and phenol in aqueous phase. J. Mol. Liq..

[35] Shrivastava A., Gupta V.B. (2011). Methods for the determination of limit of detection and limit of quantitation of the analytical methods. Chron. Young Sci..

[36] Allegrini F., Olivieri A.C. (2014). IUPAC-consistent approach to the limit of detection in partial least-squares calibration. Anal. Chem..

[37] Environmental Protection Agency (2016). Definition and Procedure for the Determination of the Method Detection Limit, Revision 2.

[38] Chen H.L., Wei G.T. (2010). The use ionic liquid as the eluent additive for HPLC separation of ionic dyes. J. Chin. Chem. Soc..

[39] Moawed E.A., Alqarni Y. (2013). Determination of azine and triphenyl methane dye in wastewater using polyurethane foam functionalized with tannic acid. Sample Prep..

[40] Shrivas K., Wu H.F. (2008). Functionalized-multiwalled carbon nanotubes as a preconcentrating probe for rapid monitoring of cationic dyestuffs in environmental water using AP-MALDI/MS. J. Sep. Sci..

[41] Wang S.-R., Wang S. (2014). Ionic liquid-based hollow fiber-supported liquid-phase microextraction enhanced electrically for the determination of neutral red. J. Food Drug Anal..

[42] Bubalo M.C., Radošević K., Redovniković I.R., Slivac I., Srček V.G. (2017). Toxicity mechanisms of ionic liquids. Arh. Za Hig. Rada I Toksikol..

[43] Miehlich B., Savin A., Stoll H., Preuss H. (1989). Results obtained with the correlation energy density functionals of becke and Lee, Yang and Parr. Chem. Phys. Lett.

[44] Check C.E., Faust T.O., Bailey J.M., Wright B.J., Gilbert T.M., Sunderlin L.S. (2001). Addition of polarization and diffuse functions to the LANL2DZ basis set for p-block elements. J. Phys. Chem. A.

[45] Ehrlich S., Moellmann J., Reckien W., Bredow T., Grimme S. (2011). System-Dependent dispersion coefficients for the DFT-D3 treatment of adsorption processes on ionic surfaces. ChemPhysChem.

[46] Pick U., Avron M. (1976). Neutral red response as a measure of the pH gradient across chloroplast membranes in the light. FEBS Lett..

[47] Banjare M.K., Behera K., Satnami M.L., Pandey S., Ghosh K.K. (2018). Self-assembly of a short-chain ionic liquid within deep eutectic solvents. RSC Adv..

[48] Chan C.C., Lee Y., Lam H., Zhang X.-M. (2004). Analytical Method Validation and Instrument Performance Verification.

[49] Kazi T.G., Shah F., Afridi H.I., Khan S., Arian S.S., Brahman K.D. (2012). A green preconcentration method for determination of cobalt and lead in fresh surface and waste water samples prior to flame atomic absorption spectrometry. J. Anal. Methods Chem..

[50] Asgharinezhad A.A., Rezvani M., Ebrahimzadeh H., Shekari N., Ahmadinasab N., Loni M. (2015). Solid phase extraction of Pb (II) and Cd (II) ions based on murexide functionalized magnetic nanoparticles with the aid of experimental design methodology. Anal. Methods.

[51] Asl Y.A., Yamini Y., Rezazadeh M., Seidi S. (2015). Electromembrane extraction using a cylindrical electrode: A new view for the augmentation of extraction efficiency. Anal. Methods.

