# Correction: Ullah et al. Ultrasound-Assisted Dispersive Liquid-Liquid Microextraction Using Deep Eutectic Solvents (DESs) for Neutral Red Dye Spectrophotometric Determination. *Molecules* 2022, *27*, 6112

**DOI:** 10.3390/molecules30132771

**Published:** 2025-06-27

**Authors:** Sana Ullah, Hameed Ul Haq, Muhammad Salman, Faheem Jan, Faisal Safi, Muhammad Balal Arain, Muhammad Shahzeb Khan, Roberto Castro-Muñoz, Grzegorz Boczkaj

**Affiliations:** 1 Department of Chemistry, University of Malakand, Chakdara 18800, Pakistan; 2 Department of Sanitary Engineering, Faculty of Civil and Environmental Engineering, Gdansk University of Technology, G. Narutowicza St. 11/12, 80-233 Gdansk, Poland; 3 School of Materials Science and Engineering, University of Science and Technology of China, Shenyang 110016, China; 4 Department of Advanced Materials Center, Faculty of Electronics, Telecommunications and Informatics, Gdansk University of Technology, G. Narutowicza St. 11/12, 80-233 Gdansk, Poland; 5 Department of Chemistry, University of Karachi, Karachi 75270, Pakistan; 6 Department of Chemistry and Technology of Functional Materials, Faculty of Chemistry, Gdansk University of Technology, G. Narutowicza St. 11/12, 80-233 Gdansk, Poland; 7 Tecnologico de Monterrey Campus Toluca, Av. Eduardo Monroy Cárdenas 2000 San Antonio Buenavista, Toluca de Lerdo 50110, Mexico; 8 EkoTech Center, Gdansk University of Technology, G. Narutowicza St. 11/12, 80-233 Gdansk, Poland

In the original publication [1], we have identified a reference error that requires correction. The reference [18] is incorrect and out of context for the given statement.

Incorrect reference [18]:

18. Vickers, N.J. Animal communication: When i’m calling you, will you answer too? *Curr. Biol.* **2017**, *27*, R713–R715. https://doi.org/10.1016/j.cub.2017.05.064.

Correct reference [18]:

18. Guo, Y.; Liu, C.; Ye, R.; Duan, Q. Advances on Water Quality Detection by UV-Vis Spectroscopy. *Appl. Sci.* **2020**, *10*, 6874. https://doi.org/10.3390/app10196874.

The authors apologize for this unintended error which happened while inserting the references using referencing software and state that the scientific conclusions are unaffected. This correction was approved by the Academic Editor. The original publication has also been updated.
