# Supplementary material for: Ultrasound-Assisted Dispersive Liquid-Liquid Microextraction Using Deep Eutectic Solvents (DESs) for Neutral Red Dye Spectrophotometric Determination
Source: Molecules. 2022 Sep 19;27(18):6112. doi: 10.3390/molecules27186112 (PMC9501489; doi:10.3390/molecules27186112)
Supplement: Supplementary file 1 [file molecules-27-06112-s001.zip › molecules-1894153-supplementary.pdf]

Supplementary material

Article

Ultrasound-Assisted Dispersive Liquid-Liquid Microextraction Using Deep Eutectic Solvents (DESs) for Neutral Red Dye Spectrophotometric Determination

Sana Ullah [1], Hameed Ul Haq [2,*], Muhammad Salman [1], Faheem Jan [3], Faisal Safi [4], Muhammad Balal Arain [5], Muhammad Shahzeb Khan [6], Roberto Castro-Muñoz [2,7] and Grzegorz Boczkaj [2,8,*]

[1] Department of Chemistry, University of Malakand, Chakdara 18800, Lower Dir, Khyber Pakhtunkhwa, Pakistan

[2] Department of Sanitary Engineering, Faculty of Civil and Environmental Engineering, Gdansk University of Technology, G. Narutowicza St. 11/12, 80-233 Gdansk, Poland

[3] School of Materials Science and Engineering, University of Science and Technology of China, Shenyang 110016, China

[4] Department of Advanced Materials Center, Faculty of Electronics, Telecommunications and Informatics, Gdańsk University of Technology, G. Narutowicza 11/12, 80-233 Gdańsk, Poland

[5] Department of Chemistry, University of Karachi, Karachi 75270, Pakistan

[6] Department of Chemistry and Technology of Functional Materials, Faculty of Chemistry, Gdansk University of Technology, Narutowicza 11/12, 80-233 Gdansk, Poland

[7] Tecnologico de Monterrey Campus Toluca, Av. Eduardo Monroy Cárdenas 2000 San Antonio Buenavista, 50110 Toluca de Lerdo, Mexico

[8] EkoTech Center, Gdansk University of Technology, G.Narutowicza St. 11/12, 80-233 Gdansk, Poland

* Correspondence: hameed.haq@pg.edu.pl (H.U.H.); grzegorz.boczkaj@pg.edu.pl (G.B.); Tel.: +48-697970303; Fax: +48-58-347-26-94

Supplementary material

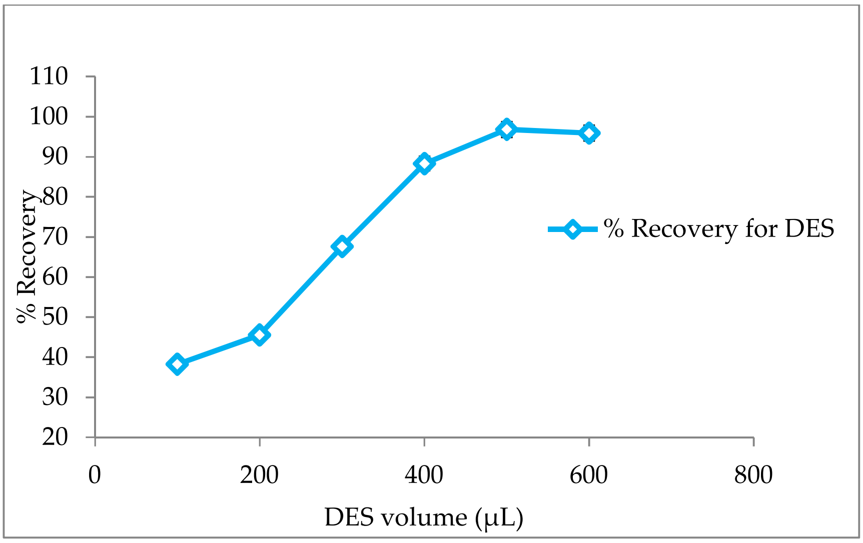

Figure S1. Optimization of DES volume, THF volume: 300µL, Sample volume: 20 mL, NR concentration: 400 µg/L.

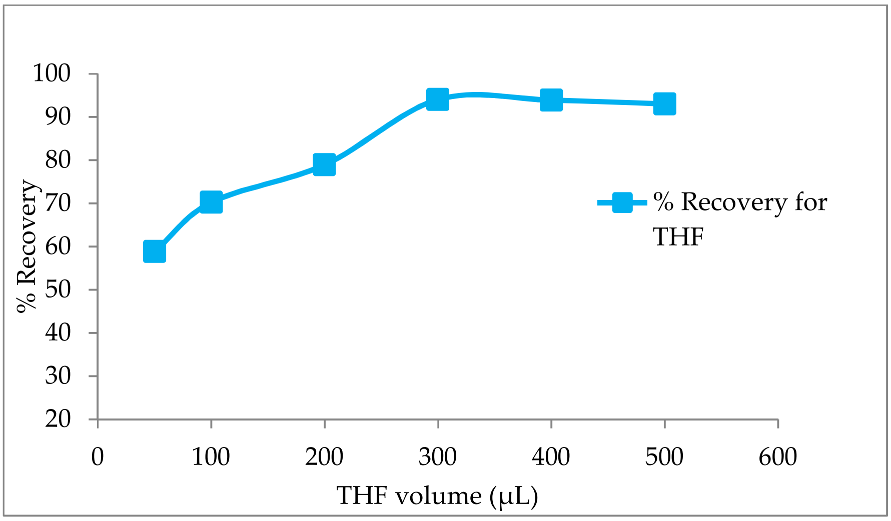

Figure S2. Optimization of THF volume, DES volume 500 µL, Sample volume: 20 mL, NR concentration: 400 µg/L

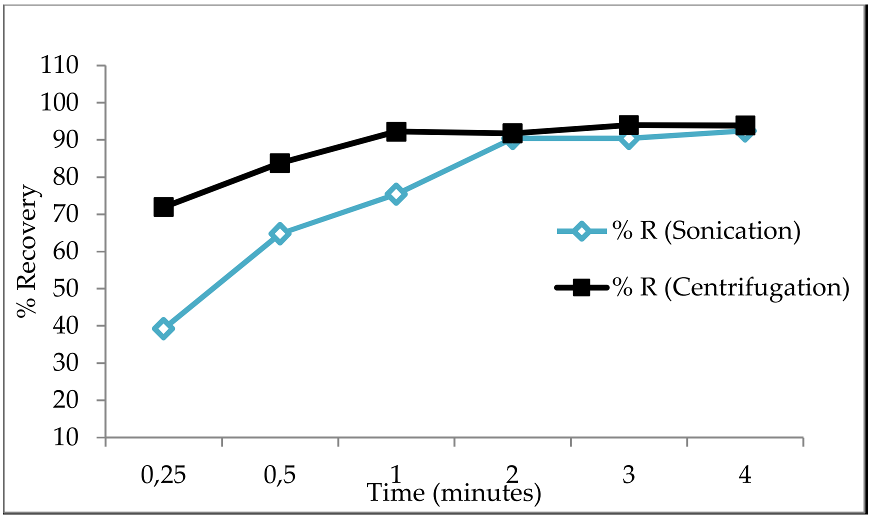

Figure S3. Time optimization for sonication and centrifugation, DES volume 500 µL, THF volume: 300µL, Sample volume: 20 mL, NR concentration: 400 µg/L

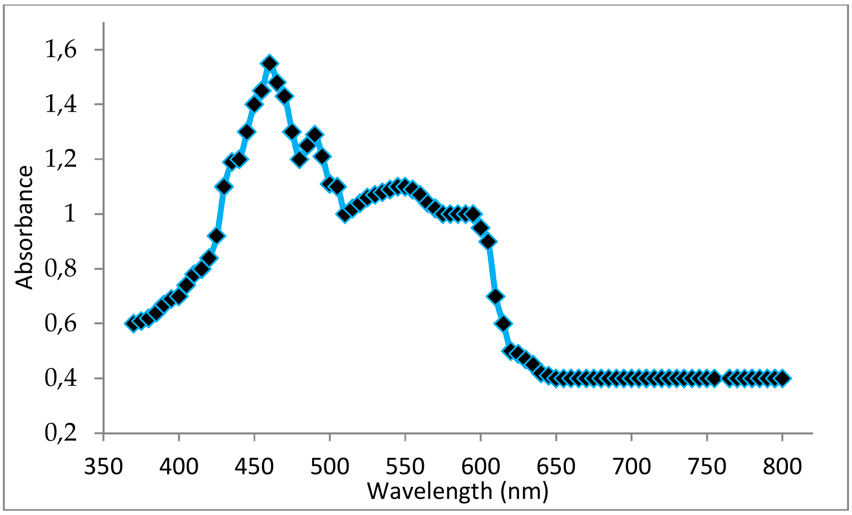

Figure S4. Selection of optimum wavelength (Lambda max) for NR quantification
